# Exosomes from *Ureaplasma parvum*-infected ectocervical epithelial cells promote feto-maternal interface inflammation but are insufficient to cause preterm delivery

**DOI:** 10.3389/fcell.2022.931609

**Published:** 2022-08-15

**Authors:** Ourlad Alzeus G. Tantengco, Lauren S. Richardson, Enkhtuya Radnaa, Ananth Kumar Kammala, Sungjin Kim, Paul Mark B. Medina, Arum Han, Ramkumar Menon

**Affiliations:** ^1^ Division of Basic Science and Translational Research, Department of Obstetrics and Gynecology, The University of Texas Medical Branch at Galveston, Galveston, TX, United States; ^2^ Biological Models Laboratory, Department of Biochemistry and Molecular Biology, College of Medicine, University of the Philippines Manila, Manila, Philippines; ^3^ Department of Electrical and Computer Engineering, Texas A&M University, College Station, TX, United States; ^4^ Department of Biomedical Engineering, Texas A&M University, College Station, TX, United States; ^5^ Department of Chemical Engineering, Texas A&M University, College Station, TX, United States

**Keywords:** extracellular vesicles, organ-on-a-chip, cervix, fetal membrane, genital mycoplasma, pregnancy

## Abstract

This study determined if exosomes from ectocervical epithelial (ECTO) cells infected with *Ureaplasma parvum (U. parvum)* can carry bacterial antigens and cause inflammation at the feto-maternal interface using two organ-on-chip devices, one representing the vagina-cervix-decidua and another one mimicking the feto-maternal interface, and whether such inflammation can lead to preterm birth (PTB). Exosomes from *U. parvum*-infected ECTO cells were characterized using cryo-electron microscopy, nanoparticle tracking analysis, Western blot, and Exoview analysis. The antigenicity of the exosomes from *U. parvum*-infected ECTO cells was also tested using THP-1 cells and our newly developed vagina-cervix-decidua organ-on-a-chip (VCD-OOC) having six microchannel-interconnected cell culture chambers containing cells from the vagina, ectocervical, endocervical, transformation zone epithelia, cervical stroma, and decidua. The VCD-OOC was linked to the maternal side of our previously developed feto-maternal interface organ-on-a-chip (FMi-OOC). Cell culture media were collected after 48 h to determine the cytokine levels from each cell line *via* ELISA. For physiological validation of our *in vitro* data, high-dose exosomes from *U. parvum*-infected ECTO cells were delivered to the vagina of pregnant CD-1 mice on E15. Mice were monitored for preterm birth (PTB, < E18.5 days). Exosomes from ECTO cells infected with *U. parvum* (UP ECTO) showed significant downregulation of exosome markers CD9, CD63, and CD81, but contained multiple banded antigen (MBA), a *U. parvum* virulence factor. Monoculture experiments showed that exosomes from UP ECTO cells delivered MBA from the host cell to uninfected endocervical epithelial cells (ENDO). Moreover, exposure of THP-1 cells to exosomes from UP ECTO cells resulted in increased IL-8 and TNFα and reduced IL-10. The OOC experiments showed that low and high doses of exosomes from UP ECTO cells produced a cell type-specific inflammatory response in the VCD-OOC and FMi-OOC. Specifically, exosomes from UP ECTO cells increased pro-inflammatory cytokines such as GM-CSF, IL-6, and IL-8 in cervical, decidual, chorion trophoblast, and amnion mesenchymal cells. The results from our OOC models were validated in our *in vivo* mice model. The inflammatory response was insufficient to promote PTB. These results showed the potential use of the VCD-OOC and FMi-OOC in simulating the pathophysiological processes *in vivo*.

## Introduction

Extracellular vesicles (EVs) are a diverse group of membrane-bound vesicles secreted by cells into the extracellular environment (van Niel et al., 2018; Doyle and Wang, 2019). EV subtypes, such as exosomes and microvesicle (MV) apoptotic bodies, are usually differentiated based upon their biogenesis, release pathways, size, content, and function (Raposo and Stoorvogel, 2013). Exosomes are the most studied and characterized EV subtype (György et al., 2011). These are round-shaped EVs surrounded by a lipid bilayer, formed by an endosomal route, and usually are 30–100 nm in diameter (Théry et al., 2002). Exosomes play a diverse role in physiologic and pathologic processes in the human body, including pregnancy and parturition (Czernek and Düchler, 2020; Menon and Shahin, 2021), where they can function as paracrine signalers in the feto-maternal tissues during pregnancy. Clinical studies have also shown the presence and accumulation of placenta-derived exosomes in maternal circulation as early as in the first trimester of pregnancy (Salomon et al., 2014; Sarker et al., 2014). Moreover, these exosomes carry inflammatory signals to the different gestational cells, which can alter the course of pregnancy and parturition (Sheller-Miller et al., 2017; Dixon et al., 2018; Sheller-Miller et al., 2019b; Monsivais et al., 2020; Tantengco et al., 2021b; Shepherd et al., 2021). Previous *in vitro* and *in vivo* studies in our laboratory have demonstrated feto-maternal and maternal-fetal trafficking of exosomes during pregnancy, and that exosomes enriched in inflammatory cargo are capable of causing labor and delivery in animal models (Sheller-Miller et al., 2016; Hadley et al., 2018; Sheller-Miller et al., 2019a; Tantengco et al., 2021b; Shahin et al., 2021).

Exosomes also play a role in intracellular communication during bacterial infections (Zhang et al., 2018; Cheng and Schorey, 2020). Exosomes can carry pathogen molecules that serve as antigens or agonists of innate immune receptors to induce host defense and immunity. It can also propagate infection and inflammation as well as mediators of immune evasion (Schorey et al., 2014; Schorey and Harding, 2016). Infection can also affect exosome biogenesis in the host cells by altering the number of exosomes generated by the host cells, the structure of the exosome membrane, and the protein cargo of the exosomes (Gioseffi et al., 2021). This phenomenon is usually observed among intravacuolar pathogens or those that reside within membrane delimited compartments in phagocytes at some time in their life cycle within mammalian hosts (Gioseffi et al., 2021). Previous studies showed that *Mycobacterium tuberculosis* and *Helicobacter pylori* were shown to hijack the exosome biogenesis pathways to package their proteins and secrete them into exosomes from host cells (Bhatnagar et al., 2007; Giri et al., 2010; Shimoda et al., 2016). This phenomenon was also observed in *Ureaplasma parvum* (*U. parvum*), a commensal bacteria in the female genital tract associated with preterm birth (PTB), a major pregnancy complication (Nishiumi et al., 2017). *U. parvum* was shown to utilize the host cellular membrane compartments, possibly to evade the host immune system and likely force the cell to package exosomes with multiple banded antigen (MBA), a virulence factor from *U. parvum*. These exosomes from infected host cells also delivered MBA to uninfected recipient cells to generate a pathologic environment (Nishiumi et al., 2017). However, the exact functional role of these MBA-containing exosomes from *U. parvum-*infected cells during pregnancy and parturition is yet to be elucidated. This is largely due to the lack of easy-to-access suitable *in vitro* models that can be used to study the functions of these exosomes.

Organ-on-a-chip (OOC) systems, *in vitro* devices typically composed of microfabricated cell culture chambers that can better mimic the physiology of organ systems in terms of their structure and functions, have been extensively developed in the past decade and have become an important tool in conducting mechanistic studies (Richardson et al., 2020a; Ingber, 2022). OOC systems of the female reproductive system, including fetal membrane (Richardson et al., 2019), feto-maternal interface (Richardson et al., 2020b; Radnaa et al., 2021; Kim et al., 2022), cervix (Khorsandi et al., 2019; Tantengco et al., 2021c), and placenta (Blundell et al., 2016; Lee et al., 2016) have also been emerging, where they have been used to study ascending infection and inflammation (Richardson et al., 2020b; Radnaa et al., 2021), transplacental transfer of compounds and drugs (Blundell et al., 2018; Pemathilaka et al., 2019), and toxicity of environmental chemicals such as cadmium (Kim et al., 2022). These OOC devices have emerged as powerful tools to conduct mechanistic studies.

This study presented here aims to determine the impact of *U. parvum* infection in the exosomes produced by ECTO cells in terms of their morphology, tetraspanin markers, and protein cargo. We also studied the role of exosomes in propagating inflammation induced by *U. parvum* in the feto-maternal interface (FMi) using our newly developed vagina-cervix-decidua-organ-on-a-chip (VCD-OOC) and our previously developed FMi-OOC (Richardson et al., 2020b). Lastly, we determined the role of exosomes in inducing PTB using a mouse model.

## Materials and methods

### Institutional review board approval

This study used immortalized cervical and vaginal epithelial cells provided by Dr. Richard Pyles (Department of Pediatrics, The University of Texas Medical Branch at Galveston, TX, United States). The cervical stromal cells were collected and immortalized from cervical tissue biopsies obtained from non-pregnant, pre-menopausal women (< 50 years old) undergoing total hysterectomy due to benign gynecological conditions at the Columbia University Irving Medical Center using an Institutional Review Board (IRB)-approved protocol (IRB-AAAI0337). The fetal membrane cells were previously collected and immortalized from placental specimens from John Sealy Hospital at the University of Texas Medical Branch at Galveston, TX, United States, following the relevant guidelines and regulations of approved IRB protocols (UTMB 11-251; University of Texas Medical Branch at Galveston).

### Human vaginal epithelial cell, ectocervical epithelial cell, endocervical epithelial cell, and cervical stromal cell cultures

Immortalized ectocervical and endocervical epithelial cells, as well as cervical stromal cells, were used in this study. These cell lines were previously validated to model lower genital tract epithelial cells (Herbst-Kralovetz et al., 2008; Tantengco et al., 2021d; Tantengco et al., 2021e). Vaginal epithelial cells (VEC) were grown in 1:1 ratio of keratinocyte serum-free medium (KSFM) supplemented with bovine pituitary extract (30 μg/ml), epidermal growth factor (0.1 ng/ml), CaCl_2_ (0.4 mM) (ThermoFisher Scientific, Cat. #37010022), and primocin (0.5 mg/ml; ant-pm-1; Invivogen, San Diego, CA, United States) and KGM™-2 Keratinocyte Growth Medium Bulletkit™ (Lonza, Walkersville, MD, United States, Cat. #C-3107). Ectocervical epithelial (ECTO) and endocervical epithelial (ENDO) cells were cultured in complete KSFM. Cervical stromal cells were cultured in Dulbecco’s modified Eagle’s medium/Nutrient Mixture F-12 (DMEM/F12; Mediatech Inc., Manassas, VA, United States) supplemented with 10% FBS (Sigma, Cat. #F2442), 50 IU/ml penicillin/50 μg/ml streptomycin (Corning, Cat. #30-001-CI), and 2.5 μg/ml amphotericin B (SigmaAldrich, Cat. #A2942), at 37°C and 5% CO_2_ environment until 80%–90% confluency was achieved.

### Human decidua and fetal membrane cell cultures

Human decidua and fetal membrane cells used in this study were isolated from placentas collected from women undergoing elective cesarean delivery at term who were not in labor and had no other complications during pregnancy (Lavu et al., 2019; Radnaa et al., 2022). These cells were previously immortalized using the PA317 LXSN 16E6E7 (ATCC^®^ CRL-2203™) and SV40 Cell Immortalization Kit. The immortalized cells were previously characterized and showed comparable morphology, cell type-specific markers, and cell signaling pathway activation as primary cells (Radnaa et al., 2022). Human amnion epithelial cells (hFM-AEC) were cultured in KSFM supplemented with bovine pituitary extract (30 μg/ml), epidermal growth factor (0.1 ng/ml), CaCl_2_ (0.4 mM), and primocin (0.5 mg/ml). Human amnion mesenchymal cells (hFM-AMC) were cultured in DMEM/F12 supplemented with 5% FBS, 50 IU/ml penicillin/50 μg/ml streptomycin and 2.5 μg/ml amphotericin B. Human decidual cells (hFM-DEC) were cultured in DMEM/F12 supplemented with 10% FBS, 50 IU/ml penicillin/50 μg/ml streptomycin and 2.5 μg/ml amphotericin B. Human chorion trophoblast cells (hFM-CTC) were cultured in DMEM/F12 supplemented 0.2% FBS, 0.1 mM 2-mercaptoethanol (Gibco, Cat. #50-114-7851), 0.5% P/S, 0.3% BSA (Gemini Bio, Cat. #700-100P, West Sacramento, CA, United States),1% ITS-X-supplement (Gibco,Cat.#51-500-056), 2 μM CHIR99021 (Sigma, Cat. #SMl1046), 0.5 μM A8301 (Sigma, Cat. #SML0788), 1 μM SB431542 (Sigma, Cat. #616464), 1.5 μg/ml L-ascorbic acid (Sigma, Cat. #A4544), 50 ng/ml EGF (Sigma, Cat. #E4127), 0.8 mM VPA (Sigma, Cat. #P6273), and 5 μM Y27632 (Millipore Sigma, Cat. #68800-05MG). All human fetal membrane cells were grown at 37°C and 5% CO_2_ environment until they reached 80%–90% confluency.

### Human THP-1 macrophage culture

THP-1 monocytes were obtained from the American Type Culture Collection (ATCC TIB-202) and were cultured in RPMI 1640 Medium (ATCC modification) (A1049101, Thermo Fisher Scientific, Watham, MA) and supplemented with 0.05 mM 2-mercaptoethanol and 10% FBS. The cells were grown until they reached a density of 1 × 10^6^ cells/ml. The cells were then centrifuged at 1,000 rpm for 5 min. THP-1 monocytes were seeded in a 48-well plate (50,000 cells per well), and they were differentiated to THP-1 macrophages by incubating them in a culture medium containing 100 mM phorbol 12-myristate 13-acetate for three days at 37°C and 5% CO_2_ environment, and grown to 80% confluency as described previously (Tantengco et al., 2021a).

### Bacterial strain and culture conditions

*U. parvum* (ATCC^®^ 700970™) was obtained from the ATCC and propagated in UMCH medium (Namba et al., 2010). A mixture of *Mycoplasma* broth base (Becton, Dickinson and Co., Baltimore, MD, Cat. # 211458) 1.47% (wt/vol), yeast extract (Becton, Dickinson and Co., Cat. #211546) 2.5% (wt/vol), horse serum (Biowhittaker, Walkersville, MD) 20% (vol/vol), urea 0.04% (wt/vol), phenol red 0.001% (wt/vol), l-cysteine hydrochloride 0.01% (wt/vol), and penicillin G 1,000 U/ml was used as the culture medium. *U. parvum* were incubated for 16–18 h to obtain titers of 1 × 10^9^–1 × 10^11^ color-changing units (CCU)/ml of viable bacteria. The corresponding amounts of *Ureaplasma* DNA were verified using a standard genesig Real-time PCR detection kit (Z-Path-U. *parvum*-std, American Research Products Inc., Waltham, MA), and amounted to 3 × 10^7^–3 × 10^8^ copy numbers/ml.

For animal studies, we used the pathogenic strain ATCC 12014 *Escherichia coli* O 55:K59(B5):H obtained from Remel Laboratory of Thermo Fisher (Thermo Fisher Scientific, Remel Products, Lenexa, KS, United States, Lot # 496291) as our positive control for PTB. This strain was used because our previous study showed that 10^11^ CFU of this *E. coli* strain induced PTB within 30 h of *E. coli* administration in the vagina of CD-1 pregnant mice (Spencer et al., 2021). The bacteria were cultured in sterile, non-selective nutrient broth (BD Biosciences), and stocks were stored in 20% glycerol at −80°C.

### *U. parvum* infection in ectocervical epithelial cells

Approximately 2 × 10^6^ ECTO cells were loaded in T75 culture flasks and allowed to grow until 80%–90% confluency (approximately 8 × 10^6^ cells). ECTO cells were infected with 1 × 10^10^ CCU/ml *U. parvum* for 4 h. The culture media containing the *U. parvum* was removed, and the ECTO cells were washed twice with sterile 1 × PBS. ECTO cells were then treated with 200 μg/ml gentamicin for 3 h to kill all remaining extracellular *U. parvum*. The culture media containing gentamicin was removed, and the ECTO cells were washed twice with sterile 1 × PBS. The ECTO cells were cultured in standard KSFM media, incubated at 37°C, 5% CO_2_, and 95% air humidity for 48 h. The culture media were collected for exosome isolation.

### Exosome isolation

Approximately 200 ml of culture media was collected from three T75 culture flasks of ECTO cells and then subjected to differential ultracentrifugation, as described previously (Sheller-Miller et al., 2017; Monsivais et al., 2020; Tantengco et al., 2021e) with some modifications. This exosome isolation protocol was validated to yield pure exosome samples devoid of cellular contaminants. Here, we performed at least three replicates for exosomes isolation. In brief, the culture media were sequentially centrifuged at 300 × *g* for 10 min and at 2,000 × *g* for 2 h to remove any cell debris using a Sorvall Legend X1R and TX-400 swinging bucket rotor (Thermo Fisher Scientific). The supernatant was collected and transferred to an Amicon® Ultra-15 100 kDa device (Merck, Cat. # UFC910024) to concentrate it to 2 ml by centrifugation at 4,000 × *g* for 30 min. The concentrated sample was then collected from the collection device (∼200–300 μl) and transferred to a microcentrifuge tube. The collected sample was filtered through a 0.8 µm filter and centrifuged at 10,000 × *g* for 30 min to remove all microvesicles. The supernatant was filtered through Nalgene Syringe Prefilter Plus filters and then ultra-centrifuged (Beckman Optima LX-80 ultracentrifuge, 70.1Ti rotor, Beckman Coulter) at 100,000 × *g* for 2 h to collect the exosomes. The supernatant was discarded while the pellet was resuspended in 100 µl of PBS and then passed through an Exo-spin™ column (Cell Guidance System LLC, MO, United States). The purified exosomes were eluted by adding 200 µl of 1 × PBS to the column. The collected exosome suspensions were stored at −80°C until analysis.

### Extracellular vesicles-Track

We have submitted all relevant data from our experiments to the EV-TRACK (https://evtrack.org/) knowledgebase (EV-TRACK ID: EV220088). The ECTO cell exosomes used in this study received a score of 56% from EV-TRACK.

### Nanoparticle tracking analysis with ZetaView for exosome quantitation

Nanoparticle tracking analysis was performed using the ZetaView PMX 110 instrument (Particle Metrix, Meerbusch, Germany) and its corresponding software (ZetaView 8.02.28; Particle Metrix). Frozen exosomes in 1 × PBS were thawed on ice. A 1: 30 dilution of the exosome sample was made with MilliQ water (Millipore Sigma, St. Louis, MO, United States). Exosomes from uninfected and *U. parvum*-infected ECTO cells (*n = 3*) were loaded into the ZetaView nanoparticle tracking analyzer, and the number of particles/ml and size distribution was counted for each sample. The machine was cleaned between samples using filtered water. The ZetaView (Particle Metrix) results were used to calculate the number of exosomes produced per cell type for the two treatment groups.

### Western blot analysis for exosome markers

The cervical exosomes were lysed using 10 × RIPA lysis buffer (0.50 M Tris pH 8.0, 1.50 M NaCl, 10% Triton X, 5% sodium deoxycholate, and 10% SDS) supplemented with protease and phosphatase inhibitor cocktail (1: 10 v/v). The lysis mixture was vortexed for 30 s, sonicated for 30 s, and kept on ice for 30 min. Protein concentrations in the prepared samples were determined using a Pierce BCA protein assay kit (Pierce, Rockford, IL, United States). The protein samples (*n* = 3), ∼1 μg, were separated using SDS-PAGE on gradient (4%–15%) Mini-PROTEAN1TGX™ Precast Gels (Bio-Rad, Hercules, CA, United States) and transferred to the membrane using a Trans-Blot Turbo Transfer System (Bio-Rad, United States). Membranes were blocked in 5% nonfat milk in 1 × Tris-buffered saline-Tween 20 (TBS-T) buffer for 2 h at room temperature. The membranes were probed with CD63 (Novus Biologicals, CO, United States, Cat. #NBP2-32830) and multiple banded antigen (MBA) (MyBioSource, Inc., San Diego CA, Cat. # MBS313152) antibodies overnight at 4°C. The membrane was incubated with an appropriate peroxidase-conjugated IgG secondary antibody for 1 h at room temperature. All blots were developed using ECL chemiluminescence reagents and a western Blotting Detection System (Amersham, Piscataway, NJ, United States) according to the manufacturer’s recommendations.

### Exoview detection of exosome tetraspanin markers

The exosomes were analyzed using the ExoView platform (NanoView, Boston, MA, United States) following the manufacturer’s procedure with modifications. ExoView allows the detection of specific cargo protein at a single-vesicle level. Briefly, 35 μl of exosomes (1 × 10^9^/ml) from uninfected and *U. parvum*-infected ECTO cells were diluted in solution A (NanoView Biosciences) and incubated on tetraspanin microarray chips placed in a 24-well plate overnight at RT. Each chip was pre-coated with CD9, CD63, CD81 antibodies, and MIgG control antibodies. Solutions and buffers provided by the manufacturer for ExoView experiments are proprietary, and their exact composition is unknown to the investigators. The following day, unbound exosomes were washed three times for 3 min using a 500 rpm shaker in solution A. Exosomes bound to the capture spots were then fixed and permeabilized with the ExoView cargo kit according to the manufacturer’s protocol. Briefly, the bound exosomes were fixed with solution C (NanoView Biosciences) for 10 min, washed as previously described (Radnaa et al., 2021), and lysed in solution D (NanoView Biosciences) for 10 min, and then washed again as previously described (Radnaa et al., 2021).

The ECTO cell exosomes were stained with exosome tetraspanin markers CD9, CD63, and CD81 antibodies diluted in blocking solution (NanoView Biosciences) for 1 h at room temperature and in the dark. The tetraspanin microarray chips were then sequentially washed three times for 5 min in solution A, in solution B (NanoView Biosciences), and five times for 5 min in Milli-Q water (ELGA) using a 500 rpm shaker. Then, the chips containing exosomes were carefully dried from the final wash step, placed on an absorbent paper, and then imaged on the ExoView R100 instrument (NanoView Biosciences) using the nScan 2.9.3 acquisition software. The size distribution, concentration, and expression of exosome tetraspanin markers were calculated using the NanoViewer 2.9.3, and the output was displayed and stored on an Excel spreadsheet.

### Exosome treatment of human THP-1 macrophages

Approximately 50,000 human THP-1 macrophages per well were plated in 48-well plates and grown overnight. The next day, the cell medium was removed, washed with 1 × PBS, and replaced with an exosome-free cell medium. Cell treatments with exosomes from uninfected and *U. parvum*-infected ECTO cells were performed by adding them to the wells. Approximately 2.2 × 10^6^ exosomes per well (low dose) and 2.2 × 10^8^ exosomes per well (high dose) from either uninfected or *U. parvum*-infected ECTO cells were added per well. The cells were incubated with the exosomes for 24 h. On completion of the treatment period, the medium was collected from each well and stored at −80°C until use.

### Enzyme-linked immunosorbent assay (ELISA) for determining inflammatory marker response

Culture medium was collected from human THP-1 macrophages after 24 h of exposure to the treatment conditions indicated above (*n = 5*). ELISA was performed to determine the concentrations of granulocyte-macrophage colony-stimulating factor (lower limit of detection = 4.7 pg/ml, GM-CSF; BD Biosciences, San Diego, CA, United States, Cat. #555126), interleukin-6 (lower limit of detection = 4.7 pg/ml, IL-6, BD Biosciences, Cat. #555220), interleukin-8 (lower limit of detection = 3.1 pg/ml, IL-8, BD Biosciences, Cat. #555244), interleukin-10 (lower limit of detection = 7.8 pg/ml, IL-10, BD Biosciences, Cat. #555157), progesterone (lower limit of detection = 47.9 pg/ml, P4, Invitrogen, Cat. #EIAP4C21), and tumor necrosis factor-α (lower limit of detection = 7.8 pg/ml, TNFα, BD Biosciences, Cat. #555212) as markers of inflammation. Standard curves were developed with duplicate samples of known quantities of recombinant proteins that the manufacturer provided. Sample concentrations were determined by relating the absorbance values that were obtained to the standard curve by linear regression analysis.

### Microfluidic vagina-cervix-decidua-organ-on-a-chip design

**FIGURE 1:**
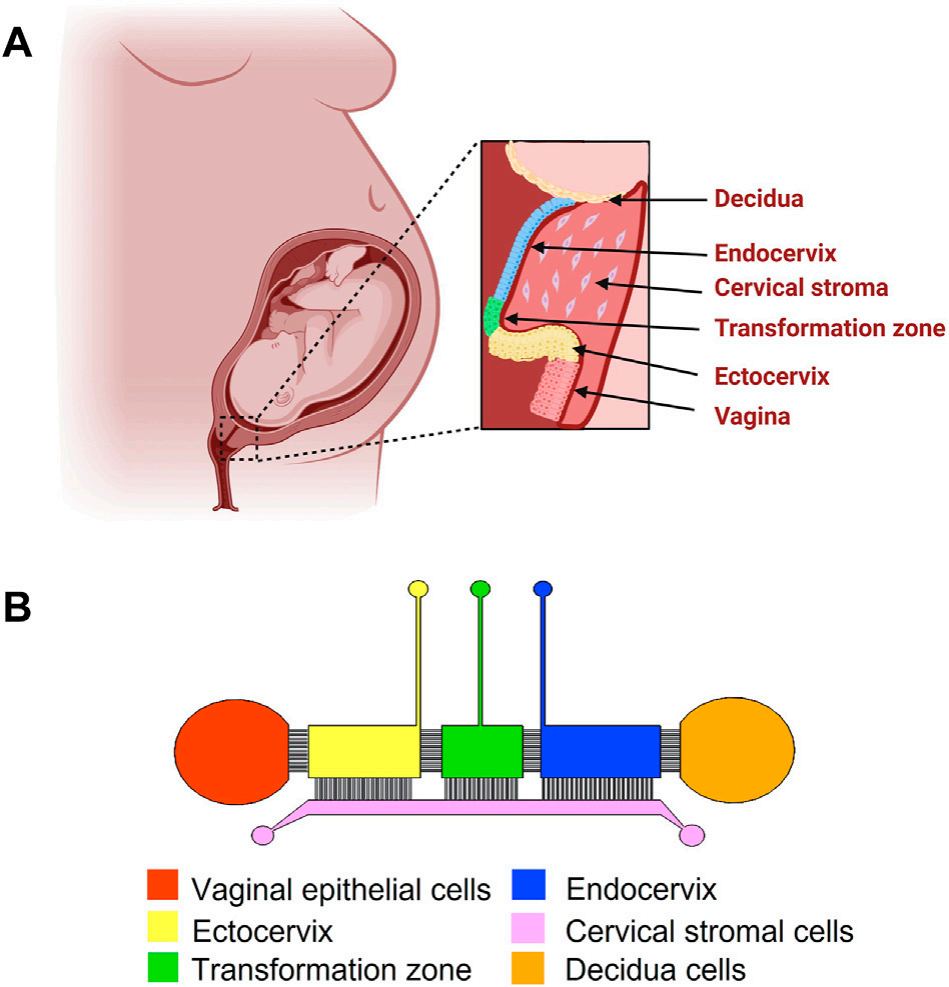
Schematic diagram of the female reproductive tract and the design of the vagina-cervix-decidua (VCD)-OOC. **(A)** An illustration of the anatomy and histology of the female reproductive tract showing the vagina, cervix, and decidua. The epithelial cells of the vagina are continuous with the ectocervix, transformation zone, and endocervix. Beneath the epithelial layers are the cervical stromal layer imbedded in collagen. During term gestation, the fetal membrane, specifically the decidua, which is its outermost layer, lies directly above the endocervix. **(B)** Schematic image of the VCD-OOC with different cell culture chambers represented by different colors and connected with each other by an array of microchannels.

The VCD-OOC device (Figure 1) was designed to model the interfaces between the human vagina, cervix, and decidua. The device is composed of six cell culture chambers mimicking the vagina, ectocervical (ECTO), transformation zone (TZ), endocervical (ENDO) epithelia, cervical stroma, and decidua layer. The five vagina-cervix epithelial-decidua chambers are interconnected by an array of 24 microchannels that are 5 μm in height, 30 μm in width, and 300 μm in length. This design allows cells to remain in their respective chambers during the cell loading process, while allowing molecular interactions (both cell-secreted molecules as well as drugs being tested) between the two chambers as well as active cell migration. This interconnected co-culture design has been previously used in our feto-maternal interface OOC (FMi-OOC) device design (Richardson et al., 2020b). The cervical epithelial chambers are connected to a single large cervical stromal chamber by a total of 72 microchannels (=5 μm in height, 30 μm in width, and 600 μm in length; 24 per chamber). These microchannels are filled with type IV collagen to mimic the basement membrane of the cervix, like what we have done previously for the FMi-OOC device (Richardson et al., 2020b; Radnaa et al., 2021; Kim et al., 2022).

### Using the vagina-cervix-decidua-organ-on-a-chip to study infected ectocervical epithelial cell exosome-induced inflammatory response in the cervicovaginal space

The exosomes produced from ECTO cells (uninfected and *U. parvum*-infected) to be used in the OOC experiments were washed with 70% ethanol for 10 min for sterilization and then rinsed three times with 1 × phosphate-buffered saline (PBS). Then, the microchannels were coated with Matrigel^®^ (Corning Matrigel^®^ Basement Membrane Matrix, DEV-free; 1:4 in KSFM) by loading Matrigel^®^ to the ECTO, TZ, and ENDO cell chambers of the VCD-OOC and applying suction pressure from the stroma cell chamber through a p1000 pipette tip attached to a vacuum system. The device was then incubated overnight at 37°C 5% CO_2_ environment. After overnight coating with Matrigel^®^, the devices were washed three times with complete KSFM before cell seeding. ECTO (80,000 cells), ENDO (80,000 cells), TZ (20,000 cells), cervical stromal (10,000 cells), decidual (50,000 cells), and vaginal epithelial cells (40,000 cells) were trypsinized and seeded into the VCD-OOC. For inflammatory marker analysis, 2.2 × 10^6^ exosomes per well (low dose) and 2.2 × 10^8^ exosomes per well (high dose) from either uninfected or *U. parvum*-infected ECTO cells were added into the ECTO chamber and allowed to propagate to the other cell culture chambers through diffusion. After 48 h, the cells and culture medium from each chamber were collected for downstream analysis.

### Using the feto-maternal interface-OOC to study infected ECTO cell exosome-induced inflammatory response in the feto-maternal interface

To complete our OOC model of ascending infection from the vagina to the fetal membranes as seen during pregnancy, we also used our previously developed FMi-OOC device. In this two-OOC devices strategy, the supernatant was collected from the decidua chamber of the VCD-OOC device (after its vaginal chamber was inoculated with exosomes produced by *U. parvum*-infected ECTO cells and cultured for 48 h) and then added to the decidua chamber of the FMi-OOC device. The FMi-OOC device was designed and manufactured as previously described in their use for modeling ascending infection in the feto-maternal interface (Richardson et al., 2020b; Radnaa et al., 2021). Briefly, hFM-AECs (300,000 cells) were seeded into chamber #1 (outermost), hFM-AMCs (40,000 cells) were seeded into chamber #2, hFM-CTCs (300,000 cells) were seeded into chamber #3, and hFM-DECs (10,000 cells) were seeded into chamber #4 (innermost). These cell ratios mimic the ratios seen in fetal membranes *in vivo*. Cells were seeded a day before the exosome experiments to ensure proper cell attachment and confluency within the device. Media (30 µl) collected from the hFM-DEC chamber of the VCD-OOC (after its vaginal chamber was inoculated with exosomes from *U. parvum-*infected ECTO cells and cultured for 48 h) and was added to the hFM-DEC chamber of the FMi-OOC and incubated for 48 h. After the incubation period, culture media was collected from each of the four chambers of the FMi-OOC for cytokine analysis.

### Supernatant analysis through inflammatory cytokine assays

The presence of cytokines granulocyte-macrophage colony-stimulating factor (GM-CSF), interleukin (IL)-6, IL-8, and IL-10 were analyzed from the cell supernatants in the VCD-OOC and FMi-OOC devices. Supernatants were collected from the reservoirs of all chambers after 48 h of treatment. Multiplexed cytokine assays were performed using a Luminex 200 (LX200-XPON-IVD, Luminex Corporation, Austin, TX, United States) apparatus. Standard curves were developed with duplicate samples of known quantities of recombinant proteins provided by the manufacturer. Sample concentrations were determined by relating the fluorescence values obtained to the standard curve by linear regression analysis.

### Carboxyfluorescein succinimidyl ester staining of cervical exosomes for IVIS imaging

Carboxyfluorescein succinimidyl ester (CFSE) was used to stain the ECTO cell exosomes for subsequent imaging to monitor their transport to the uterine cavity *via* whole tissue imaging with IVIS. ECTO cell exosomes were stained with either 100 µM of CFSE for 30 min at 37°C, and excess dye was washed three times with 1 × PBS. The cervical exosomes were transferred to the Amicon^®^ ultra-15 100 KDa device to concentrate them to 200 μl by centrifugation at 4,000 × g for 10 min. Three doses of 4.5 × 10^8^ CFSE-stained ECTO cell exosomes were administered vaginally to E15 mice, 30 min apart per dose. Mice were sacrificed 24 h after the exosome administration. The whole intact uterus, including the embryos, was removed and transferred on ice to the Biomedical Imaging Facility at UTMB and imaged with the IVIS Spectrum CT *In Vivo* Imaging System (PerkinElmer, Waltham, MA, United States) to determine the localization of exosomes in the uterus.

### Physiologic validation of the OOC experiments using a mouse model of PTB

For physiological validation of the *in vitro* OOC data, we tested if *U. parvum*-infected ECTO cell exosomes can cause PTB in CD-1 mice. All animal procedures were approved by the Institutional Animal Care and Use Committee (IACUC) at UTMB. Timed pregnant CD-1 mice were purchased from Charles River Laboratories (Houston, TX, United States) and received on gestational day 14 (E14) and were then housed in a temperature- and humidity-controlled facility with 12 h:12 h light and dark cycles. On E15, pregnant mice were anesthetized with inhalation of isoflurane and subjected to vaginal administration of bacteria suspension using 200 ml pipette tips. A volume of 40–60 μl was chosen based on our previous study (Spencer et al., 2021) showing vaginal administration without leakage.

The following treatment groups were used for PTB monitoring: 1) vaginal delivery of sterile 1 × PBS; 2) vaginal delivery of 2 ×10^6^ (low dose) and 2 ×10^8^ (high dose) exosomes from uninfected ECTO cells; 3) vaginal delivery of 2 ×10^6^ (low dose) and 2 ×10^8^ exosomes from *U. parvum*-infected ECTO cells. A total of three doses of each treatment was given 30 min apart; 4) vaginal delivery of 1 × PBS (negative control); and 5) vaginal delivery of 10^11^ CFU *E. coli* (positive control). The animals were continuously monitored using Wansview cameras (Shenzhen Wansview Technology Co., Ltd, Shenzhen, China) to determine the delivery timing. Delivery on or before E18.5 contributing to developmentally immature pups was considered PTB.

### Statistical analyses

All data were analyzed using Prism 7 software (GraphPad Software, La Jolla, CA, United States). The Shapiro–Wilk test for normality was conducted to check for the normality of the data. The student *t*-test was used to compare results with two means. Ordinary one-way analysis of variance followed by Tukey’s multiple comparison test was used to compare normally distributed data with at least three means. The Kruskal–Wallis test with Dunn’s multiple comparison test was used for data that were not normally distributed. Asterisks denote *p* values as follows: *p < 0.05, **p < 0.01, ***p < 0.001, and ****p < 0.0001.

## Results

### *U. parvum* infection alters protein cargo and downregulates tetraspanin markers in exosomes produced by ectocervical epithelial cells

**FIGURE 2:**
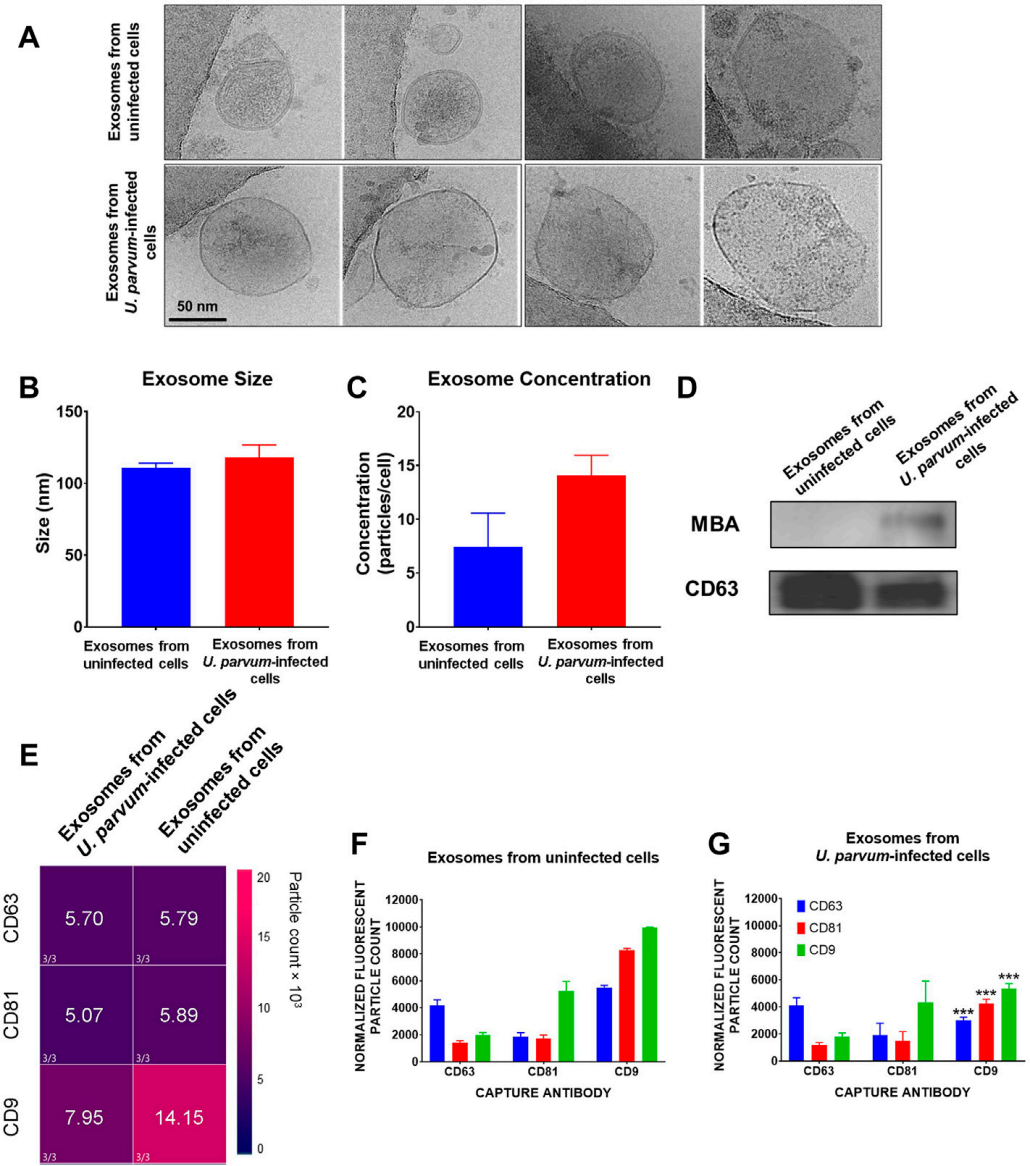
Characterization of exosomes from ECTO cells infected with *U. parvum*. **(A)** Cryo-electron micrograph of cervical exosomes shows circular exosomes. Scale, 50 nm. **(B,C)** Nanoparticle tracking analysis of cervical exosomes did not show significant differences in exosome concentration (particles/cells) and exosome size of exosomes from uninfected and *U*. *parvum*-infected ECTO cells. **(D)** All cervical exosomes expressed the exosomes marker, CD63. However, only the exosomes from infected cells expressed MBA *via* western blot. Error bars represent median ± SD. *n* = 3 technical replicates. **(E,G)** ExoView analysis of exosomes from uninfected vs., *U*. *parvum*-infected ectocervical epithelial cells. **(E)** Heat map showing the normalized expression of tetraspanin markers in ECTO cell exosomes. **(F,G)** ExoView counts of immunofluorescent stained positive exosomes for all tetraspanin markers detected. The data are presented as the means ± SEM. ***p < 0.001.

Cryo-EM analysis showed that control ECTO cell exosomes, regardless of treatment, showed circular morphology (Figure 2). Control ECTO cell exosomes were 110.8 ± 7.926 nm in diameter, while *U. parvum*-infected ECTO cell exosomes were 118.2 ± 17.25 nm in diameter. The lipid bilayer membrane can be seen distinctly in the uninfected ECTO cell exosomes (Figure 2A top image) but was somehow obliterated in the *U. parvum*-infected ECTO cell exosomes (Figure 2A bottom image). Exosome size was further confirmed by Zetaview analysis (Figures 2B,C), where *U. parvum* infection did not significantly change the size (∼ 100 nm). *U. parvum* infection resulted in higher ECTO cell exosome production; however, it did not reach statistical significance. The concentrations were 7.437 ± 5.426 exosomes per cell (normal ECTO cell exosomes) and 14.04 ± 3.315 exosomes per cell (*U. parvum*-infected ECTO cell exosomes). Western blot analysis showed that the exosomes from infected and uninfected ECTO cells expressed the tetraspanin marker CD63 (Figure 2D). However, as expected, only the exosomes from *U. parvum*-infected ECTO cells expressed MBA (Figure 2D). Exoview analysis showed that *U. parvum* infection significantly downregulated all the tetraspanin markers tested, i.e., CD9 (*p* < 0.001), CD63 (*p* < 0.001), and CD81 (*p* < 0.001) compared to exosomes from uninfected ECTO cells (Figures 2E,G).

### Exosomes from *U. parvum*-infected ectocervical epithelial cells deliver multiple banded antigen to endocervical epithelial cells

**FIGURE 3:**
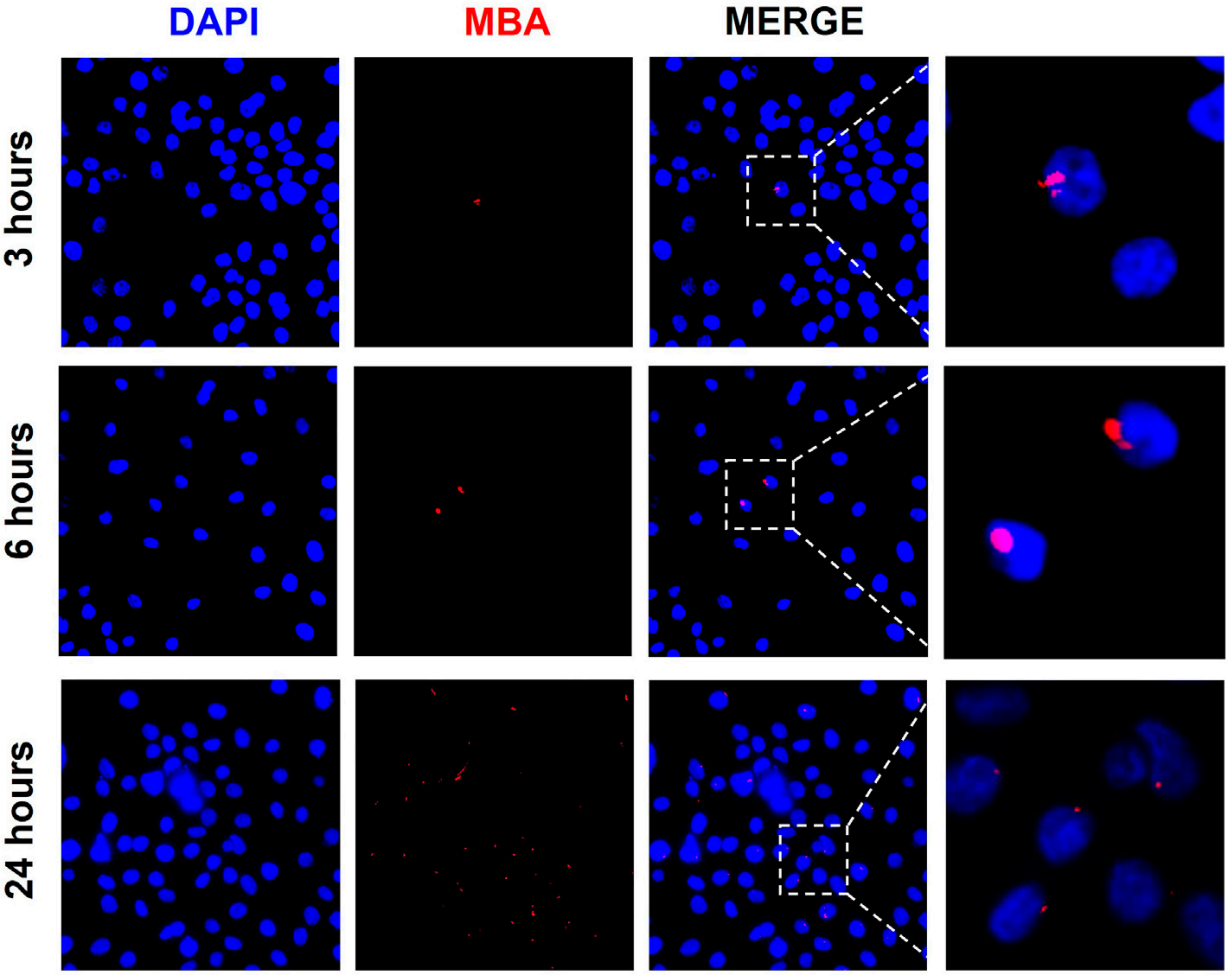
Exosomes from *U. parvum*-infected ECTO epithelial cells deliver MBA to ENDO cells. Immunocytochemical staining of multiple banded antigen (MBA), a lipoprotein and known virulence factor of *U. parvum,* in ENDO cells treated with exosomes from *U. parvum*-infected ectocervical epithelial cells for 3, 6, and 24 h. Red–MBA. Blue–DAPI.

To determine the role of exosomes in carrying *U. parvum* virulence factor MBA to other cells, we treated ENDO cells with exosomes from *U. parvum*-infected cells and determined the expression of MBA in the recipient ENDO cells. As early as 3 h post-exosome treatment, ENDO cells expressed MBA. We observed the persistence of MBA in ENDO cells after 24 h post-treatment with exosomes. However, while there were more ENDO cells with MBA staining, the fluorescence intensity were diminished when compared to the 3 h and 6 h treatments (Figure 3).

### Exosomes from *U. parvum*-infected ectocervical epithelial cells do not promote massive inflammation in maternal and fetal cells

**FIGURE4:**
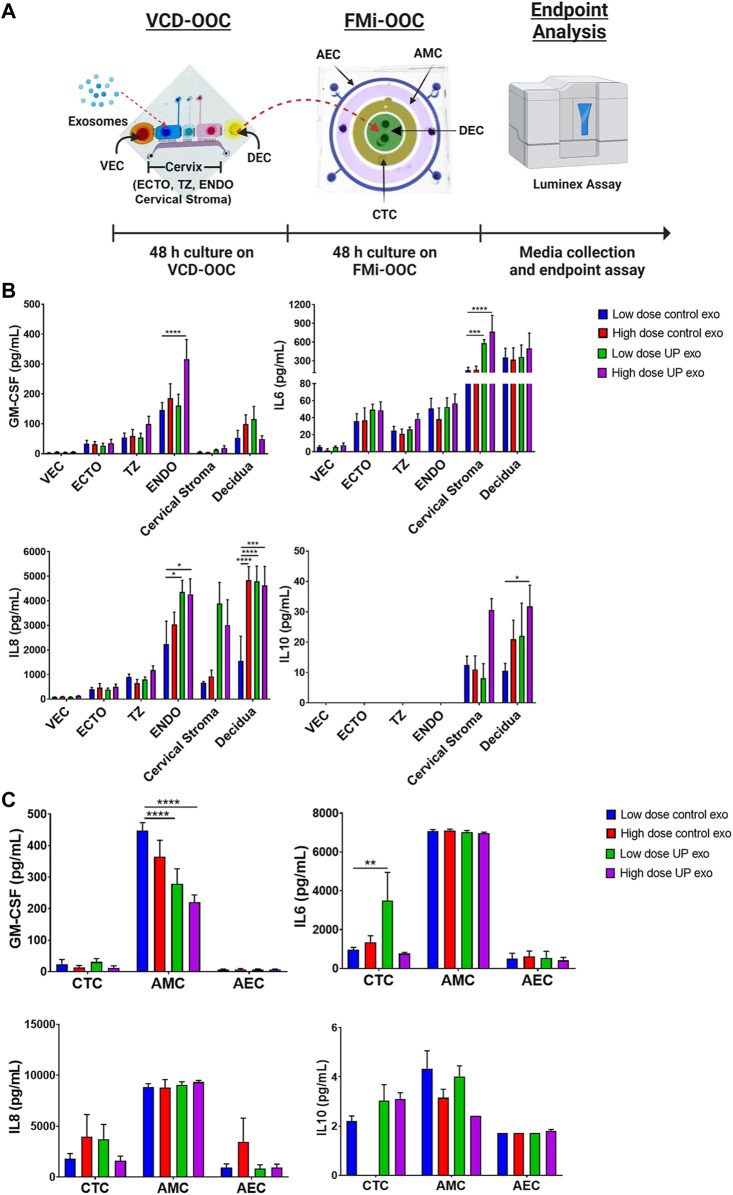
Inflammatory effects of exosomes from ECTO cells infected with *U. parvum* in the VDC-OOC and FMi-OOC. **(A)** We added low dose (2.2E6 particles) and high dose exosomes from uninfected and *U. parvum*-infected ECTO cells in the cell culture chamber for ectocervical epithelial cells. After 48 h, the cell culture media from the decidual cell chamber in the VCD-OOC was transferred to the decidual cell chamber of the FMi-OOC. Cell culture media from each chamber in the VCD-OOC and FMi-OOC were collected and subjected to Luminex assay to determine the inflammatory cytokine (GM-CSF, IL-6, IL-8, and IL-10) production by the cells in the VCD-OOC and FMi-OOC. **(B)** Inflammatory cytokine (GM-CSF, IL-6, IL-8, and IL-10) production by vaginal epithelial cells (VEC), ectocervical (ECTO), transformation zone (TZ), endocervical (ENDO) epithelial cells, cervical stromal cells, and decidual cells in the vagina-cervix-decidua-on-a-chip (VCD-OOC) after 48 h of exosome treatment. **(C)** Inflammatory cytokine (GM-CSF, IL-6, IL-8, and IL-10) production by human chorion trophoblast cells (hFM-CTC), amnion mesenchymal cells (hFM-AMC), and amnion epithelial cells (hFM-AEC) in the feto-maternal interface organ-on-a-chip (FMi-OOC) after 48 h of exosomes treatment. *n >* 4 biological replicates. Error bars represent mean ± SEM. *p < 0.05; **p < 0.01; ***p < 0.001; ****p < 0.0001.

To investigate the effects of ECTO cell exosomes on the FMi cells, we used the VCD-OOC and FMi-OOC devices in sequence to create an OOC model of the female reproductive tract that represents the organs from vagina to fetus (Figure 4A). The utility of each of these models has been reported previously in modeling ascending infection using live *U. parvum and* lipopolysaccharide (LPS) (Richardson et al., 2020b), to test the impact of fetal cell exosomes (Radnaa et al., 2021) and environmental toxicants cadmium in causing inflammation (Kim et al., 2022) in feto-maternal interface cells.

First, low and high doses of exosomes from uninfected and *U. parvum*-infected ECTO cells were loaded into the ECTO cell chamber of the VCD-OOC and allowed to propagate throughout the cell layers in the VCD-OOC for 48 h. After the treatment, the cell culture medium from each cell chamber was subjected to ELISA to determine their inflammatory cytokine levels. The VCD-OOC experiment showed that low-dose exosomes from *U. parvum*-infected ECTO cells increased the IL-6 level in the cervical stromal cell chamber (*p <* 0.001), and the IL-8 level in the ENDO cell chamber (*p* < 0.05) as well as in the hFM-DEC cell chamber (*p* < 0.0001) (Figure 4B). No significant changes in the GM-CSF and IL-10 levels were observed. On the other hand, high-dose exosomes from *U. parvum*-infected ECTO cells increased the GM-CSF and IL-8 levels in the ENDO cell chamber (*p* < 0.0001) and the IL-6 level in the cervical stromal cell chamber (*p* < 0.0001). This treatment also increased the IL-8 level (*p* < 0.001) and the IL-10 level (*p* < 0.05) in the DEC chamber. Exosomes from *U. parvum*-infected ECTO cells promoted moderate inflammation in cervical and decidual cells.

Next, to further test the impact of ascending infection and inflammation in the vaginal chamber on the fetal side, the media containing exosomes and inflammatory mediators from the DEC chamber of the VCD-OOC was transferred to the DEC chamber of the FMi-OOC device. Stimulation by exosomes from *U. parvum-*infected ECTO cells significantly increased IL-6 levels in the CTC chamber (*p* < 0.01) and significantly decreased GM-CSF levels in the AMC chamber (*p* < 0.0001) (Figure 4C). However, this treatment did not affect the anti-inflammatory cytokine levels in CTC, AMC, and AEC chambers. These results showed that exosomes from *U. parvum*-infected ECTO cells only promoted mild inflammation in the CTC layer but did promote inflammation in the amnion layer of the fetal membrane. To note, the cytokine response was heterogeneous in both OOC experiments, and cell type-specific cytokine responses were seen.

### Exosomes from *U. parvum*-infected ectocervical epithelial cells promote pro-inflammatory responses in human macrophages

**FIGURE 5:**
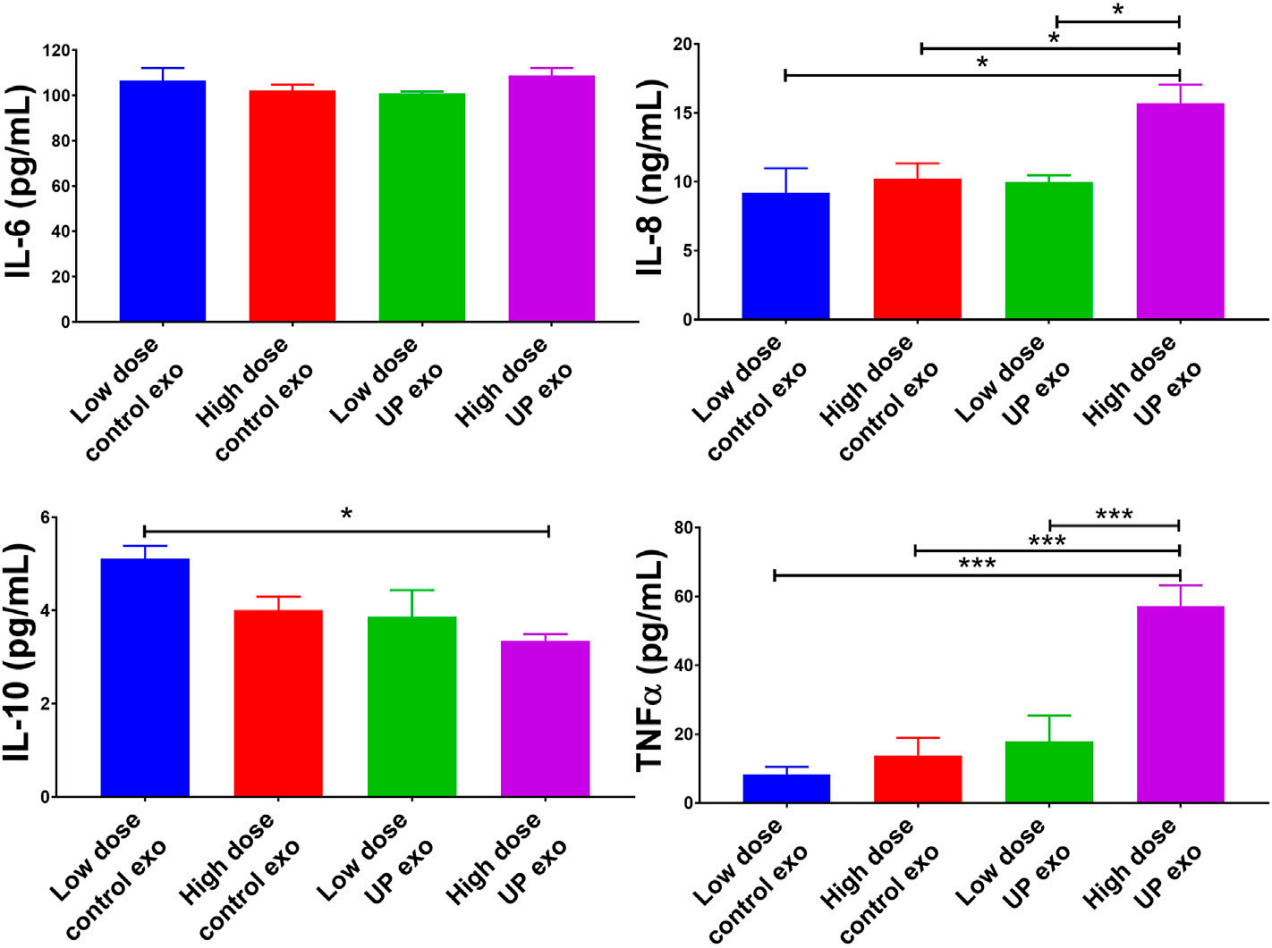
Exosomes from ECTO cells infected with *U. parvum* promote pro-inflammatory responses in human macrophages. Pro-inflammatory cytokines human IL-6, IL-8, and TNFα, and anti-inflammatory cytokine IL-10 levels in culture medium collected from THP-1 macrophages after 24 h treatment with exosomes from uninfected vs. *U. parvum-*infected ECTO cells. Error bars represent mean concentration ± SEM, *n* = 5 technical replicates. *p < 0.05; **p < 0.01; ****p < 0.0001.

To check if exosomes from *U. parvum*-infected ECTO cells can also promote inflammation in immune cells, we treated THP-1 macrophages with these exosomes. After 24 h, the culture media were subjected to ELISA to check for the levels of inflammatory cytokines. Exosomes from *U. parvum*-infected ECTO cells significantly increased the IL-8 and TNFα (pro-inflammatory cytokines) levels and significantly reduced the IL-10 (anti-inflammatory cytokine) levels (Figure 5).

### Ectocervical epithelial cell-derived exosomes delivered vaginally reached the uterus of pregnant mice

**FIGURE 6:**
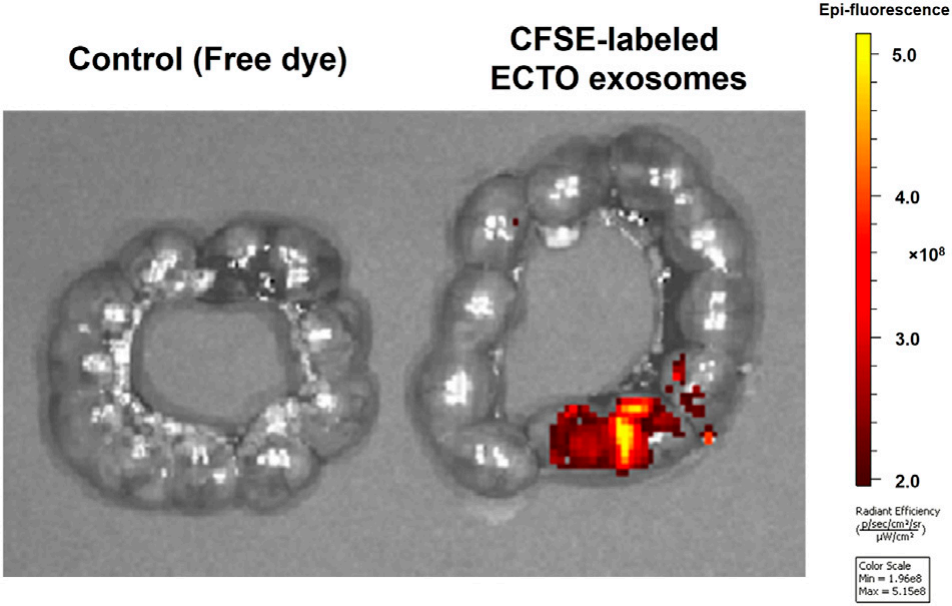
Localization of ECTO cell-derived exosomes in the uterine cavity. CFSE-stained ECTO epithelial cell exosomes in the uterine cavity capture via *ex-vivo* IVIS imaging. Uteri were collected and imaged with IVIS 24 h after vaginal administration of three doses of 4.5 × 10^8^ ECTO cell exosomes to E15 pregnant mice.

We performed vaginal administration of exosomes produced by ECTO cells into E15 pregnant mice to determine whether the ECTO cell exosomes can reach the fetus. We checked their localization in the uterine cavity after 24 h by IVIS. As can be seen in Figure 6, ECTO cell exosomes reached the uterine cavity after 24 h, where the exosomes were primarily localized in the proximal portion of the uterus near the cervix (Figure 6).

### Exosomes from *U. parvum*-infected ectocervical epithelial cells do not promote preterm birth but cause neonatal death

**FIGURE 7:**
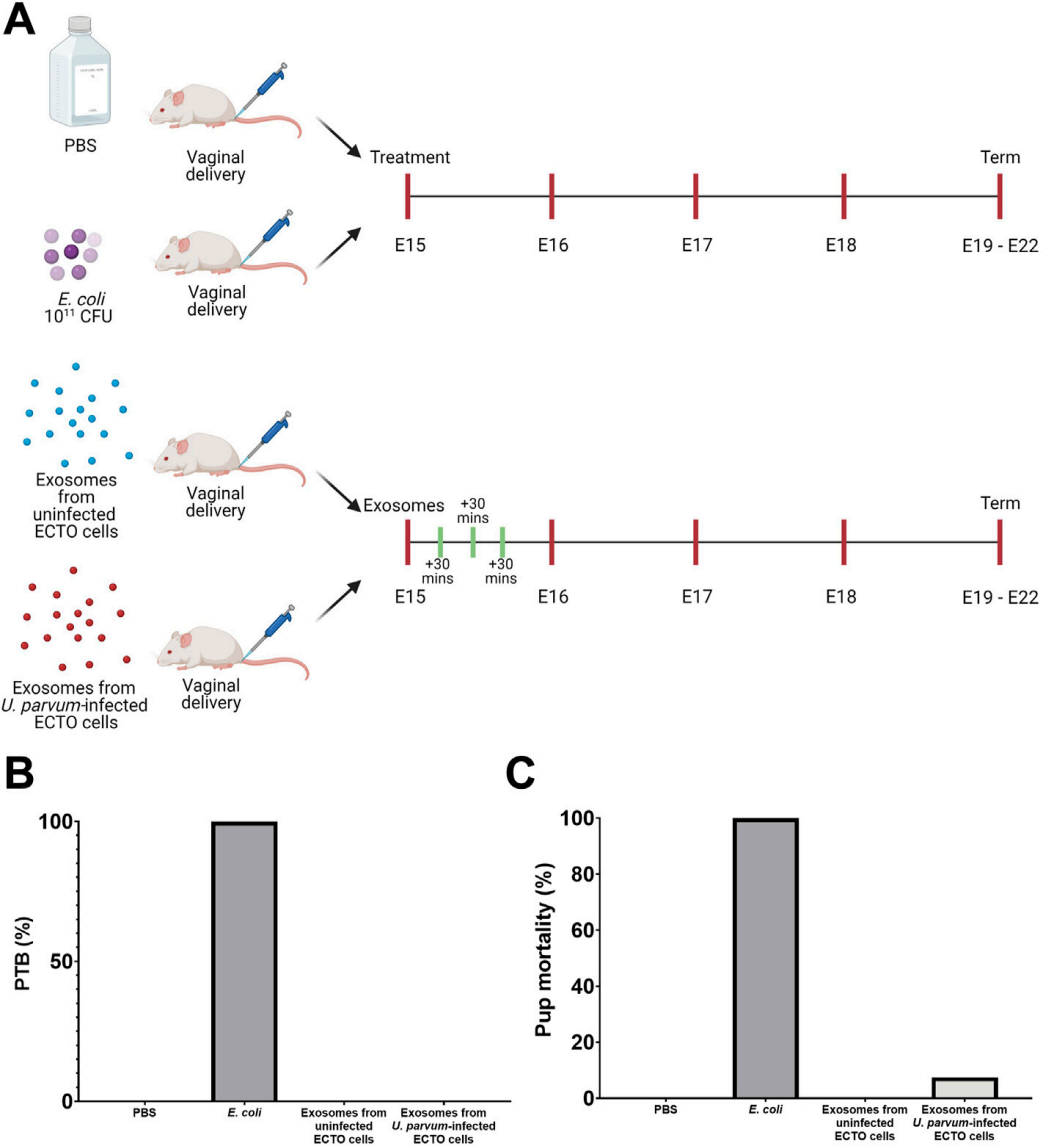
Preterm birth and neonatal death from exosomes from *U. parvum-*infected ECTO cells in CD-1 mice. **(A)** Schematic diagram of the *in vivo* experimental design. E15 pregnant mice were injected vaginally with exosomes from uninfected and *U. parvum*-infected ectocervical epithelial cells. **(B)** Preterm birth rate and **(C)** pup mortality rate at birth after exosome treatment. For all groups, *n* ≥ 5 pregnant mice.

For physiological validation of the OOC data, exosomes from uninfected and *U. parvum*-infected ECTO cells were inoculated vaginally in CD-1 mice. PTB was monitored, and pup mortality was noted (Figure 7A). As expected, PBS (negative control) neither caused PTB nor pup mortality, while ascending infection with *E. coli* (positive control) promoted 100% PTB and pup mortality (Figures 7B,C). Exosomes either from uninfected or *U. parvum*-infected ECTO cells did not induce PTB in CD-1 mice. However, CD-1 mice treated with exosomes from *U. parvum*-infected ECTO cells showed a mild impact on pup mortality (7.5%) (Figures 7B,C).

## Discussion

In this study, we used integrated OOC models of the vagina-cervix-decidua interface (VCD-OOC) and the feto-maternal interface (FMi-OOC) to study the impact of exosomes produced by *U. parvum*-infected ECTO cells on the female reproductive tract during pregnancy. Our results showed that the exosomes contained *U. parvum*-derived antigens and promoted inflammation in cervical and decidual cells, but not in fetal membrane cells such as chorion trophoblast cells, amnion epithelial cells, and amnion mesenchymal cells. The absence of a massive inflammatory response in the fetal membrane cells was interpreted as an indicator that this exosome treatment may be insufficient to cause PTB. Our physiologic validation using CD-1 pregnant mice indeed showed that vaginal delivery of exosomes from *U. parvum*-infected ECTO cells was insufficient to cause PTB. The presence of moderate inflammation in the cervix-decidua interface and the absence of inflammation in the feto-maternal interface may explain the lack of PTB in our *in vivo* model. Maternal inflammation alone without a massive fetal inflammatory response may not be enough to promote preterm birth (Gomez et al., 1998; Romero et al., 2006). These results highlight the utility of OOC models in predicting pregnancy outcomes in a mouse model.

*U. parvum* is associated with adverse pregnancy outcomes such as spontaneous PTB and preterm prelabor rupture of the membranes (Taylor-Robinson, 2007; Kacerovský et al., 2009). However, the mechanistic manifestation of this association and pathways of *U. parvum*-mediated adverse events have not been well established. This knowledge is critical in providing proper intervention. Prior reports (Noda-Nicolau et al., 2016; Polettini et al., 2018; Pavlidis et al., 2020) have shown that *U. parvum* alone in the absence of other virulent pathogens (e.g., *E. coli*) does not cause PTB and that the inflammatory response is more balanced in the intrauterine cavity. In the presented study, we showed that intercellular pathogen like *U. parvum* may propagate their pathogenicity *via* infected cell exosomes.

This study showed that exosomes carry *U. parvum* virulence factors such as MBA from the host cell to other uninfected cells. Moreover, we showed the functional effects of these exosomes on the feto-maternal cells using the integrated VCD-OOC and FMi-OOC model and on immune cells using THP-1 macrophages. Our results showed that exosomes from *U. parvum*-infected ECTO cells could promote a pro-inflammatory environment in the cervical epithelial cells, stromal cells, and macrophages. However, inflammation was non-specific, showed heterogeneity (cell type-dependent), and promoted more balanced pro and anti-inflammatory responses in the fetal membrane cells. Furthermore, our *in vivo* studies showed that exosomes from *U. parvum*-infected ECTO cells were insufficient to induce PTB and resulted in only minimal pup mortality (7.5%).

Based on this new insight, we propose that live *U. parvum* infection or exosomes from cells infected with *U. parvum* carrying bacterial antigens (i.e., MBA) can promote mild inflammation in the cervicovaginal and feto-maternal interface cells, but is insufficient to promote PTB. *In vitro* and *in vivo* studies showed that *Ureaplasma* spp. Infection in the lower genital tract does not always promote PTB unless there is a concomitant infection with a more virulent pathogen or in cases where the cervical barrier is compromised (i.e., patients with cervical insufficiency, chronic cervicitis, short cervix, or previous cervical surgery) (Tantengco and Menon, 2022).

One of the interesting findings from our study is the downregulation of tetraspanin markers of exosomes (CD9, CD63, and CD81) produced by *U. parvum*-infected ECTO cells. Tetraspanins are small transmembrane proteins usually found in exosomes. They act as molecular scaffolds connecting different proteins for signal transduction (Lu et al., 2017). Tetraspanins CD9, CD63, and CD81 regulate protein cargo sorting into exosomes (Chairoungdua et al., 2010; Nazarenko et al., 2010; van Niel et al., 2011). Depletion of tetraspanins such as CD81 has been reported to reduce specific proteins associated with CD81, including MHC molecular, ICAM-1, and Rac, in the exosomes (Nazarenko et al., 2010). Tetraspanins also play a fundamental role in determining the exosome’s target cell and uptake (Schorey and Bhatnagar, 2008; Andreu and Yáñez-Mó, 2014). This process is essential because uptake of exosomes can alter the cellular processes in recipient cells. Exosome uptake in recipient cells may regulate gene expression or activate signaling cascades (Schorey and Bhatnagar, 2008). The downregulation of tetraspanins in exosomes from *U. parvum-*infected cells may indicate alterations in the exosome biogenesis, protein cargo sorting, and selectivity of target cells. These changes may be responsible for the distinct morphological changes in the membranes of exosomes from cells infected with *U. parvum*. Moreover, downregulation of tetraspanin may also be involved in packaging MBA, a virulence factor specific to *Ureaplasma* spp., into the exosomes from infected host cells. Based on these data, we hypothesized that *U. parvum* might control exosome biogenesis in ectocervical epithelial cells to promote its survival, and also propagate infection and inflammation into nearby cells through the exosomes. The number of exosomes released is not impacted by *U. parvum* infection. This suggests that the likely impact of infection in ECTO cells is on the cargo packaging.

The results from our *in vitro* 2D monoculture, OOC model experiments, and *in vivo* studies showed that exosomes from *U. parvum*-infected cells could carry MBA to neighboring cells and promote inflammation. Several bacteria, such as *Mycobacterium tuberculosis*, *Salmonella* typhimurium, and mycoplasmas, have been shown to exploit the exosome pathway to disseminate their antigens and promote an inflammatory response in adjacent and distal cells (Bhatnagar et al., 2007; Yang et al., 2012; Schorey et al., 2014; Nishiumi et al., 2017). Exosomes from *S. typhimurium*-infected THP-1 cells contained lipopolysaccharide. Moreover, exosomes from THP-1 cells infected with *M. tuberculosis* increase TNF-α and IL-12 production, and have been shown to recruit macrophages and neutrophils *in vivo* (Bhatnagar et al., 2007; Giri et al., 2010). On the other hand, exosomes released from mycoplasma-infected tumor cells promote activations of splenic inhibitory B cells and increase splenocytes’ anti-inflammatory cytokine release (Yang et al., 2012). Lastly, a previous study showed that *U. parvum* infection could exploit the host exosome biogenesis pathways to evade the host immune system (Nishiumi et al., 2017). *U. parvum* can be internalized into HeLa cells, evade lysosomal degradation, and survive in the perinuclear region for at least 14 days. Exosomes isolated from HeLa cells with intracellular *U. parvum* were shown to contain MBA (Nishiumi et al., 2017). Based on this evidence and our current evidence, exosomes act as carriers of antigens for immune cell activation and modulation of immune cell functions.

Exosomes can also impact the outcome of infection by interacting with and modifying immune cells, as well as promoting inflammation, matrix deposition, and vascular permeability (Schorey et al., 2014; Rodrigues et al., 2018). Our animal model studies showed that vaginal delivery of multiple doses of exosomes from *U.* parvum-infected cells was insufficient to cause PTB. However, it is still unclear whether exosomes containing a component of the pathogens are released in sufficient quantities to promote local immune responses. More studies are needed to determine whether exosomes from infected cells promote or inhibit host immunity.

This study and our prior reports showed that exosomes from the fetal side could traffic to the maternal side and vice versa (Sheller-Miller et al., 2016; 2019a). The role of exosomes in carrying bacterial components have several implications in pregnancy and parturition. Although *U. parvum*-infected cell-derived exosomes are not overtly immunogenic in the uterine and fetal membrane tissues or induce PTB, these data formulated several hypotheses. First, pathogens do not need to cross the placenta or break through the cervical mucus plug to elicit an inflammatory or immune response in the fetus. In the setting of ascending infection from the lower genital tract to the amniotic cavity, we hypothesize that infected cells in the lower genital tract may release exosomes that can carry bacterial antigens to uterine tissues, fetal membranes, and even the fetus. Similarly, we hypothesize that exosomes from infected cells outside the female reproductive tract are released into the maternal circulation and cross the placental barrier to elicit an immune response in the placenta as well as in the fetus.

Another implication of the role of exosomes in carrying bacterial components, including bacterial nucleic acid materials, and delivering them to other tissues, including sterile sites such as the placenta, is that they can be mistakenly identified as placental microbiome (Aagaard et al., 2014; Cao et al., 2014; Blaser et al., 2021). Recent studies showed that healthy placentas do not display a distinct microbiome (de Goffau et al., 2019; Wei et al., 2020; Sterpu et al., 2021). It was reported that the amplified placental microbiome from previous studies was obtained from contamination during the experimental procedure (Gschwind et al., 2020). Moreover, previous studies were unsuccessful in culturing bacteria from the placenta. Based on this evidence, it may be possible that exosomes play an important role in transporting bacterial components to the placenta. Exosomes from other body sites with high microbial biomass such as the lower genital tract, gastrointestinal tract, and oral cavity may end up in the placenta. These exosomes may carry bacterial components that can be detected in microbiome studies (Kaminski et al., 2019).

### Strengths and limitations of the presented study

The strengths of our study include the use of both OOC and animal models in investigating the functional role of exosomes during *U. parvum* infection on the maternal and fetal cells during pregnancy. Our integrated VCD-OOC and FMi-OOC models allowed us to recreate a model of the vagina-cervix-decidua integrated with the fetal membranes, which were used in our exosome trafficking studies. However, the OOC models still have several limitations. Our OOC models do not have immune cells, components that are essential in inflammatory and immune responses in the feto-maternal interface. However, we performed stimulation assays using 2D culture of THP-1 cells to check the functional effects of exosomes in immune cells. Our VCD-OOC model also lacked the endocrinologic stimulation (i.e., estrogen and progesterone) seen in *in vivo* physiology during pregnancy. Lastly, since we are only interested in pregnancy outcomes, we did not collect data on the inflammatory responses of different maternal (e.g., cervix and uterus) and fetal (e.g., fetal membrane, fetus, and placenta) tissues in our animal studies. Despite these limitations, our model still allowed us to better understand the functional effects of exosomes from *U. parvum*-infected ECTO cells on the feto-maternal interface using *in vitro* OOC and animal models.

## Conclusion

*U. parvum* infection altered the tetraspanin expression and protein cargo of ECTO cell exosomes. These exosomes contained *U. parvum*-derived antigens and induced a moderate inflammatory response in the cervix and fetal membrane cells. The use OOC devices showed that exosomes from *U. parvum*-infected ECTO cells alone cannot elicit a massive inflammatory response in the feto-maternal interface cells. These results corroborated with the *in vivo* mice experiments which showed that these exosomes were insufficient to cause PTB. These exosomes did not induce PTB and only promoted minimal pup mortality in *in vivo* studies. These results suggest that *U. parvum* can alter the exome biogenesis machinery, protein cargo sorting, and release in cervical cells. Whether these effects propagate infection and inflammation to promote or inhibit host immunity remains to be elucidated. Further studies are needed to help us better understand the roles of exosomes from infected cells in the immune response under physiological and pathological conditions during pregnancy.

## Data Availability

The original contributions presented in the study are included in the article/Supplementary Material, further inquiries can be directed to the corresponding authors.
